# Enabling food system innovation: accelerators for change

**DOI:** 10.1016/j.gfs.2023.100738

**Published:** 2024-03

**Authors:** Philip Thornton, Daniel Mason D'Croz, Cody Kugler, Roseline Remans, Heather Zornetzer, Mario Herrero

**Affiliations:** a Department of Global Development, College of Agriculture and Life Sciences, Cornell University, Ithaca, New York, USA; b Cornell Atkinson Center for Sustainability, Cornell University, Ithaca, New York, USA; c Agricultural Economics and Rural Policy Group, Wageningen University and Research, Wageningen, the Netherlands; d Bioversity International, Heverlee, Belgium; e Glocolearning, Genk, Belgium

**Keywords:** Technology, Food system, Accelerator gap, Adoption, Impact

## Abstract

It is widely accepted that current food systems are not on a trajectory for achieving the Sustainable Development Goals by the end of the decade. Technological innovation will have a considerable role to play in different parts of the food system; many promising options exist or are in the pipeline, some of which may be highly disruptive to existing value chains. Scaling up the innovations required, at the same time as protecting those who may lose out in the short term, will require a strong enabling environment. Here we apply an existing framework of eight change accelerators to six case studies of historical agricultural innovation. We estimated the degree to which each accelerator had been addressed at some stage in the innovation process, as a measure of the gap between what was needed and what was achieved. For the innovations that are being taken to scale and widely utilized, these accelerator gaps are small. Uptake of other innovations is stalled, and for these we found large gaps for one or more of the eight accelerators. Impactful innovation processes address all eight change accelerators at some point, with different phasing of the accelerators depending on the nature of the technology and on the impact pathway being pursued. This simple framework, when used in combination with narratives of uptake based on theories of change and impact pathways, may provide an effective means of screening future innovation processes to help prioritize and guide investment that can lead to more resilient, sustainable and equitable food systems.

## Highlights

• Food system innovation will be needed to meet food security and environmental goals.
• A framework of eight innovation accelerators was applied to six historical case studies.
• The impactful case studies addressed all accelerators at some time during the innovation process.
• The framework can help screen pipeline innovations to guide future investment.

## 1 Introduction

Many reports have been published in recent years about the necessity of transforming global food systems, to the point where this is hardly in doubt (Webb et al., 2020). The challenge is formidable, in the light of the climate emergency (IPCC, 2022), pandemics such as Covid-19 that highlight food system vulnerabilities and interconnections (Dasgupta and Robinson, 2022), and the enormous pressures being put on natural resources (Hendriks et al., 2021).

Several questions arise in formulating a response to this challenge: who is going to produce the food needed to provide healthy and equitable diets for a global population of between 9 and 10 billion people by 2050 (Adam, 2021)? What will future consumption patterns look like? How is this food to be produced in a way that protects the environment and keeps humankind within a safe operating space? As Giller et al. (2021) note, there is bewildering diversity in farming systems around the world, and even more diversity in food systems that span multiple value chains from inputs to consumers and beyond. Given the scale of the challenge, regardless of who will be producing the food of the future and how, technological innovation will have a considerable role to play in different parts of the food system in a wide variety of contexts. Many promising options exist or are in the pipeline, some of which may be highly disruptive to existing value chains (Barrett et al., 2022; Herrero et al., 2021).

For innovations to be widely taken up, and for benefits to be realised on development and human health outcomes at scale, a strong enabling environment will be required that can accelerate the changes needed. Here we briefly describe an existing framework made up of eight of these change accelerators and then test its utility to examine six case studies of historical agricultural innovation. We discuss the results of this exercise and the possibility of using this simple framework to empower future innovations in food systems.

## 2 Accelerators of innovation

The literature on taking innovation to scale is large and rapidly expanding, and many frameworks have been proposed (see Lam et al. (2020) for several examples). Food system transformation is a type of innovation that may involve fundamental changes in the system itself (values, regulation, policies, markets, governance) as well as in its different elements (technologies, infrastructure, know-how): for technological change to occur at scale, a wide range of social and institutional factors and conditions may be needed to enable their deployment (Hall and Dijkman, 2019). What emerges from this perspective are eight key building blocks to accelerate technological and systemic innovation in food systems. These are summarised in Herrero et al. (2020), along with some examples in Herrero et al. (2021), as follows:

1 **Building trust amongst actors in the food system:** developing shared vision and values, so there is some consensus and support for the changes that are being proposed, making decision-making transparent, and working towards high-level agreements about the ways in which the regulatory environment may need to be modified for ensuring appropriate environmental, health and safety standards. Given the environmental and ethical issues surrounding food production and consumption, these agreements on vision and values will be critical for legitimizing what may be highly disruptive innovations around food production and consumption.
2 **Transforming mindsets:** promoting acceptance of what may be highly technological interventions and different ways of producing and handling food and feed. This may be necessary because most people have deeply engrained biological, psychological and cultural relationships to food, which in some contexts may act as barriers to social acceptance of new technology.
3 **Enabling social licence and stakeholder dialogue:** ensuring responsible innovation through engagement with stakeholders across society, so that new or different technologies are developed and implemented in as transparent a way as possible.
4 **Ensuring stable finance**, by encouraging green and socially responsible public and private financing that does not reinforce existing inequalities, and by piloting alternative, creative funding mechanisms to promote innovation to address social and environmental objectives.
5 **Designing market incentives** to help spread the costs and risks associated with innovation, including developing targeted fiscal and trade policies such as taxes and subsidies to ensure a viable initial market to more rapidly achieve economies of scale, or improving the costing of externalities (undesirable environmental, social or health impacts that are not reflected in market prices) at source to increase the early competitiveness of new technology that might otherwise not be taken up.
6 **Changing policies and regulations** so that innovation, new technologies and industries are appropriately supported and supervised, through streamlining environmental regulations and health and safety standards, and by reducing economic and bureaucratic constraints to technological adoption and diffusion, for example.
7 **Safeguarding against undesirable effects** through monitoring the impacts of innovation and making corrections where needed; this may require setting up independent regulatory bodies to supervise new industries and transparently enforce standards and regulations, or it may require encouraging enhanced environmental, social and governance criteria disclosure and sustainable development goal reporting, for example.
8 **Developing transition pathways** that shed light on how change can be effected through time: which are the actions, and when do they need to be implemented, that may be needed for national and regional transition pathways to move towards desired futures, ensuring that those disadvantaged by change can also benefit from the fruits of innovation.

## 3 Historical case studies

We applied the innovation accelerator framework outlined above to six case studies of agricultural technology innovation, to test its effectiveness in being able to explain differences between innovations in being implemented and taken up at scale. Although the choice of case study was somewhat arbitrary, together the six, organized into three pairs, span a range of different types of food system innovation and different rates of uptake and scale of impact. The first pair of case studies involve innovations to combat vitamin A deficiency (VAD), but with markedly different rates of uptake. The second pair are innovations related to animal health and nutrition, again with different rates of uptake. The third pair are innovations relying on communications technologies (mobile telephony and TV) that are providing management information to farmers in different ways. Brief descriptions of these innovations follow.

### 3.1 Biofortified orange-fleshed sweet potato (OFSP)

The process of developing and scaling OFSP varieties that combine large increases in beta-carotene levels with drought tolerance is described in Low and Thiele (2020). It involved a wide range of activities related to the technical, organizational, leadership, and institutional environment, from the emergence of the idea (1991–1996) through a scaling phase in 15 countries under a major institutional innovation (2015-mid-2019), the Sweet potato for Profit and Health Initiative (SPHI). It was recognised early on that in combatting VAD in this way, breeding breakthroughs would have to be coupled with improved access to OFSP varieties and education to build awareness about the nutritional and health benefits to improve the adoption, production and consumption of OSFP among rural households. Significant investment was directed to advocacy and educational campaigns promoting household consumption of OFSP and associated value-chain development in countries where sweet potato is either the staple crop or an important secondary staple (Lidder and Dijkman, 2019). Huey et al. (2022) delineate several impact pathways for OFSP using Mozambique as an example, including direct purchase and consumption by urban dwellers; indirect consumption by women and children of OFSP given to neighbours by households in the intervention group; direct consumption by farm households producing the crop; and OFSP consumption by school children. Biofortified OFSP varieties have reached more than 6 million households in sub-Saharan Africa, with strongly positive economic (Pemsl et al., 2022) and health benefits (Low et al., 2017).

### 3.2 Golden rice (GR)

Varieties of rice with increased seed content of vitamin A were produced via genetic modification in the late 1990s (Bongoni and Basu, 2016). This innovation was originally developed by scientists in a public institution (IRRI, the International Rice Research Institute) and the rights were transferred to a private multinational biotechnology organisation (Syngenta), who then donated all legal rights to the Humanitarian Board to allow freedom-to-operate in lower- and middle-income countries. From early in its development, this innovation was opposed by various environmental groups, essentially because of the belief that transgenic crops are inherently dangerous and/or unacceptable (Moghissi et al., 2016). GR continues to be affected by high levels of conflict and political contestation around the introduction and diffusion of biotechnology in agriculture. GR was finally approved in mid-2021 by the Philippines authorities for use as food. The country has been ahead of the pack: it was the first Asian country to approve a biotech crop for planting by farmers (Bt maize) as animal feed in 2000. So far, it remains unclear how many people will plant, buy, and eat GR. On the consumer side, De Steur et al. (2022) indicate that some consumers react positively to golden rice, although it will need to become a cost-effective component of broader nutrition strategies that are tailored to different contexts. From a producer's perspective, GR will need to be commercialised and possibly its production incentivised, which may not be without its own challenges (Glover et al., 2020).

### 3.3 East Coast fever vaccination (ECF ITM)

Work on the development of an effective vaccine to control the tick-borne livestock disease East Coast fever (ECF) started more than 40 years ago (Perry and Dijkman, 2019). The innovation process was long and complex, though it resulted in an effective vaccine that works by infecting animals with just enough live pathogen to trigger a protective immune response at the same time as treating the animal by injecting an antibiotic. The vaccine provides life-long immunity after a single inoculation. Once the science had been developed and tested, a public-private partnership, GALVmed (www.galvmed.org), was set up to provide vaccines and other animal health products that could be purchased by small-scale livestock producers at market prices: donor funding to assist with product and market development, and the private sector to implement and roll-out at scale. The ECF infection and treatment (ITM) vaccine has provided protection for over 1.5 million cattle in eleven countries of East and southern Africa and benefited 150,000 farming households (Nene et al., 2021).

### 3.4 Fodder banks

In the late 1970s the International Livestock Centre for Africa (ILCA) and national research partners developed and promoted the concept of planting small areas of forage legumes to help alleviate the lack of dry-season feed for ruminant animals in West Africa. The innovation involved fencing off about 4 ha of a farmer's land and planting *Stylosanthes* or other forage legumes, which could then be used for strategic feeding during the early dry season. After two or three seasons, the fodder bank could be replaced by an unfertilized sorghum or maize crop that provided yields equivalent to fertilization with up to 45 kg nitrogen per ha because of the nitrogen previously fixed by the legume (Tarawali, 1991). After the crop, the area could be reconverted to a fodder bank via seed reserves in the soil. By the late 1990s, some 27,000 adopters had been identified, growing forage legumes on about 19,000 ha in 15 countries of the region (Elbasha et al., 1999). Although work on fodder banks at ILCA (now ILRI) ended in 1993 (Duncan et al., 2020), some work persisted in the region, including the use of *Stylosanthes* and other species in improved fallows for soil improvement and weed suppression in addition to their use as livestock feed (Tarawali et al., 1999) and in rehabilitating degraded farmland (Amole et al., 2021).

### 3.5 Participatory Integrated Climate Services for Agriculture (PICSA)

This innovation is an approach, developed in 2011, built around participatory tools that enable trained farmers to use climate information to improve their decision making (Clarkson et al., 2022). The approach provides participatory tools to enable trained farmers to make informed decisions for their individual contexts (Clarkson et al., 2022). Scaling has been achieved via a training-of-trainer approach and facilitation, in the case of Rwanda, one of the countries in which PICSA has been rolled out, in partnership with four local faith-based NGOs and the Rwandan Meteorological Agency. Radio listening clubs were launched in 2018 that carry regular daily weather forecasts and climate service education programs. Some 106,000 farmers in 27 of Rwanda's 30 districts have now been trained to understand climate information and incorporate it into their decision-making. Most participating farmers have changed their agricultural or livelihood management in response, and many perceive improvements in their confidence as farmers and in household food security and income (Hansen, 2021). The impact pathway for PICSA in Rwanda was notable for being modified during the project through the addition of the new partnerships that were found to be needed to develop and deliver localised, relevant information (Nowak et al., 2021). Overall, some 200,000 farmers in 23 countries have been trained in the PICSA approach, and evidence from seven countries shows that farmers have made beneficial changes to the ways in which they manage crops, livestock and other livelihood enterprises (Clarkson et al., 2022).

### 3.6 Shamba Shape-Up (SSU)

Shamba Shape-Up is a reality ‘make-over’ TV program about agricultural practices, technologies and strategies, broadcast in English and Kiswahili to Kenyan farmers with the aim of improving the livelihoods and incomes of farm households and the longevity of farms. Produced by the Mediae Company and running since 2010, it now reaches 10 million people per episode (Areal et al., 2020). Through partnering with a range of agricultural research institutions, many different technologies and practices have been featured. SSU has a free mobile back-up system “iShamba”, a farmer support platform allowing viewers to follow up with the SSU team to receive further information on the topics aired and ask questions of trained agronomists using SMS, phone calls or WhatsApp. By 2014, the net impact on Kenya's GDP was estimated to be USD 24 million, mostly arising from improved dairy and maize production as a result of farmers watching SSU and implementing changes (AECF, 2015; Clarkson et al., 2018). Viewers reported other impacts too: improved food security and nutrition, more confidence in their management ability, enhanced social status, and the reinvestment of increased income in other, off-farm, livelihood activities. The impacts on women farmers were found to be relatively higher than on men farmers (AECF, 2015). SSU is a cost-effective and efficient way of influencing agricultural practices and opens up two-way communication channels via SMS between farmers, program producers and the scientists who contribute to each episode (Clarkson et al., 2018).

The six case studies are summarised in Table 1 with respect to several characteristics, described in the table footnote. The impacts reported are quantitative where that information exists, otherwise qualitative estimates are provided, along with the information sources used for each case study.

**Table 1:** Characteristics of six historical technological innovations.

Characteristic	Combatting Vitamin A Deficiency		Animal Health & Nutrition		Communications Technologies	
	Orange Flesh Sweet Potato (OFSP)	Golden Rice (GR)	East Coast Fever Infection and Treatment (ECF ITM)	Fodder Bank (FB)	Participatory Integrated Climate Services for Agriculture (PICSA)	Shamba Shape-Up (SSU)
Stage of Readiness	Moving to scale	Gaining traction (possibly)	Moving to scale	Moving to scale	Moving to scale	Moving to scale
Intensity of disruption	Low	Low	Low	Low to moderate	Low	Low
Where in the food system does it act?	Value-added products; agricultural inputs; consumers	Agricultural inputs, primary production practices	Livestock producers, inputs and production practices	Livestock producers: inputs and production practices	Agricultural inputs	Small-scale producers: inputs and production practices
How does it act?	Replaces one crop/crop variety with another	Consumers: dietary addition/substitution	Reduces animal losses, increases productivity	Increases livestock and crop production	Improves seasonal decision making	Changes farmer behaviour to increase crop and livestock production and productivity
Key features of the innovation	Upscaling of research product through significant investment by philanthropic foundations in solving system and market failures at local levels; nutrition education at the community level	GR Humanitarian Board allowing public research institutions in lower-income countries free access to proprietary technologies and seed at no cost to smallholder farmers	A solution ignored for decades. Commercialisation only achieved through a public-private partnership, GALVmed	A working solution to the dry-season feed constraint, with knock-on benefits of nitrogen provision to subsequent annual crops	Simple tools that farmers can use, building on historical climate data and weather forecasts; training in their use; training of trainers coupled with radio broadcasts to achieve scale	Balance of entertainment and education, using widely-available communication media (TV and SMS) for 2-way communications, supplying appropriate information based on solid research to small-scale farmers
Environmental Impact	Limited: drought tolerance as a co-benefit	Limited, though opponents cite biodiversity concerns	Limited: possible reductions in acaricide residues in water courses and in water use	Medium: benefits of fixed nitrogen to subsequent annual crops and soil properties; limited GHG mitigation benefits	Moderate: increased adaptive capacity of famers (e.g., more efficient input use)	Depends on the practice change, but likely moderate via small improvements in the efficiency of resource use
Health Impact	High: decreasing VAD prevalence in target populations	Potentially high: decreasing VAD prevalence in target populations	High: decreasing ECF impacts in target cattle populations	Low-moderate: increased provision of animal source foods to livestock-owning households	Low-moderate, via impacts on enhanced food security	Moderate, via increased food availability at the household level
Economic Impact	Moderate: longer-term benefits via better health and decreasing prevalence of blindness	Potentially moderate: longer-term benefits via better health and decreasing prevalence of blindness	Moderate: reducing animal mortality rates and boosting incomes and food security in target populations, though at relatively high cost	Moderate: increased liveweight gains during the dry season and increased crop yields, boosting incomes/food security	Moderate, via reducing input costs in poor seasons and increasing outputs in good seasons	Moderate, via increases in farm income from raised production and/or productivity
Sociocultural Impact	Low-moderate: sweet potato often considered a “woman's crop” and there may be some gender effects on the inter-household sharing of vines	Uncertain: possible effects on power relations, gender and equity	Moderate: distribution of livelihood impacts may not be uniform, wealthier households & men benefiting more than poorer households & women	Low: though some evidence exists of gender-specific roles in the provisioning of livestock feed	Equity: both men and women reported increased incomes and food security. Gender: women reported benefits including increased ability to cope and increased confidence in planning	Benefits have been demonstrated for both men and women, with one study finding disproportionate benefits for the women
Impact to date	Now expanding well beyond the original project domain	Few producers or consumers have yet adopted golden rice (and to date only in the Philippines)	Potential for sub-sector wide impact. Over 1.5 million doses administered in eastern and southern Africa	Uptake has been very slow over 30 years, although exact status currently is unknown	Expanded to several countries with significant economic (and possibly social) returns	SSU reaches 10 million people per episode, changes adding to Kenya's GDP. Potential to reach a significant proportion of 3.6 (Kenya) and 1.3 (Zambia) million farmers
Information sources	Lidder and Dijkman (2019), Low and Thiele (2020), de Brauw et al. (2013), Huey et al. (2022), Pemsl et al. (2022)	Hays and Hall (2019), Bongoni and Basu (2016), Moghissi et al. (2016), De Steur et al. (2022), Glover et al. (2020), Mueller and Flachs (2022)	Perry and Dijkman (2019), Jumba et al. (2020), Nene et al. (2021), Toye et al. (2020), Homewood et al. (2006), Bishop et al. (2020)	Tarawali (1991), Duncan et al. (2020), Tarawali et al. (1999), Amole et al. (2021), Elbasha et al. (1999), Mohamed-Saleem and von Kaufmann (1995)	Clarkson et al. (2022), Clarkson et al. (2022), Hansen (2021), Nowak et al. (2021), Gumucio et al. (2020), Thornton et al. (2023)	Clarkson et al. (2018), Areal et al. (2020), AEC Fund (2015)

Characteristics:

• Stage of readiness: is the innovation at the stage of being an idea, a prototype, gaining traction, moving to scale, or mainstreamed. Five of the case-study innovations are moving to scale at different speeds, and one is hovering between the prototype and gaining traction stages for reasons explained in the text.

• Intensity of disruption: what is the level of disruption that the innovation has been causing to existing production systems or value chains, from low to high.

• The part of the food system on which the innovation acts, such as an input or production practice.

• The “mode of operation” of the innovation: does it replace one input with another, or does it modify farmer behaviour, for example.

• Environmental impact: a brief assessment of the environmental impact that the innovation has; does it affect greenhouse gas emissions or land use, for example.

• Health impact: a brief assessment of the impact the innovation may have on human health, such as increasing household food availability or decreasing VAD, for instance.

• Economic impact: a brief assessment of the economic impact the innovation may have, such as reducing costs or increasing productivity and farm income.

• Are there sociocultural impacts associated with uptake of the innovation: for example, are there benefits for women, or are the benefits being captured mostly by wealthier households.

• Impact to date: a summary statement of the impact the innovation has had to date, with respect to the number of beneficiaries reached.

## 4 Contribution of the change accelerators to the impact of each innovation

The discussion in this section is organized around the different accelerators, using comparisons of the case studies to illustrate the different contributions made by each accelerator to the dissemination, uptake (or otherwise) and impact of each innovation. Our estimate of the strength of the contribution of each of the eight change accelerators to the six historical innovation processes to date are shown in Annex Tables A1–A6. We used a qualitative contribution scale ranging from 0 (none) to 5 (large). The contributions of each accelerator were subjectively determined for each innovation by the author team, using available literature that described the scaling process to date.

These contributions are summarised across all innovations in Table 2. The eight accelerators are clustered in Table 2, recognizing that their importance may change considerably depending on the stage reached in the innovation process. Building trust, enabling social license and transforming mindsets (accelerators 1–3) will usually be the sine qua non for any innovation process to proceed; in Table 2, these are clustered as “laying the groundwork”. If “early adoption” is to happen, appropriate finance will need to be available as well as market incentives, where these do not exist (accelerators 4 and 5). For “moving to scale”, it may be necessary to address relevant policies and regulations; undesirable effects will need to be safeguarded against through monitoring, and transition pathways will need to be developed for large-scale expansion (accelerators 6–8). Although these clusters are likely to overlap in any innovation process, which itself may be anything but linear, they are useful for highlighting the changing roles of the accelerators along the innovation impact pathway. Each pair of related case studies presents contrasting examples of these roles.

**Table 2:**
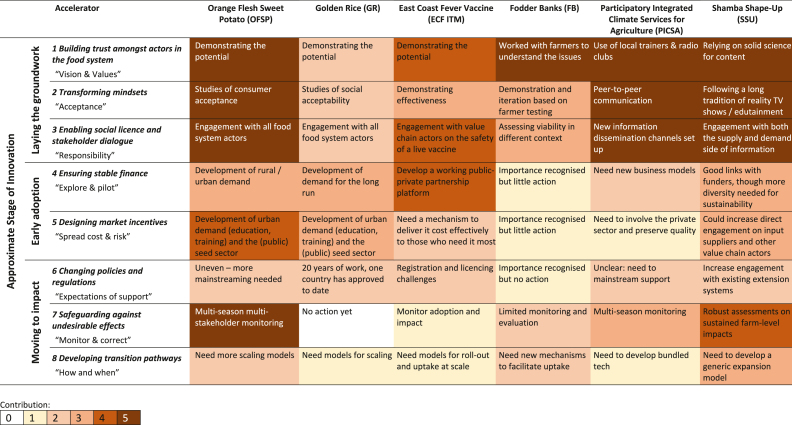
Accelerators of change of six historical technological innovations. Cells show the contribution to date made by each accelerator to the implementation of each innovation. Contributions are scored as follows: 0, no contribution to date; 1, small; 2, small to moderate; 3, moderate; 4, moderate to large; 5, large. The text in each cell indicates what has been done and/or what still needs to be done with respect to future uptake. Details for each innovation are shown in Annex Annex Table A1, Annex Table A2, Annex Table A3, Annex Table A4, Annex Table A5, Annex Table A6.

### 4.1 “Laying the groundwork” accelerators: OFSP and GR

The generic theory of change for biofortified crops set out by Huey et al. (2022) highlights impact pathways that revolve around direct purchase and consumption, informal seed transfers from adopting households to neighbours, formal consumption in feeding programs, and own-consumption by adopting households. The uptake of OFSP has examples of each of these impact pathways in southern Africa. There have been significant improvements in the Vitamin A status of people in intervention areas and beyond (de Brauw et al., 2019; Brouwer and Tedesco, 2019; GCNF, 2019). The groundwork for this impact was laid early in the innovation process: the potential of OFSP was demonstrated to a wide range of stakeholders, helping to build trust among and promoting dialogue between the key value chain actors. Studies were undertaken to gauge consumer acceptance of OFSP varieties with respect to the traits that were of importance to consumers, helping to foster acceptance of new varieties when in due course they appeared in the market (Low and Thiele, 2020). As Table 2 indicates, OFSP could achieve even greater impact with more consistent governmental and policy support for its uptake across the countries of sub-Saharan Africa (SSA) (accelerator 6). While OFSP can be spread through informal channels by farmers, involving the formal seed sector to increase the availability of quality planting material at the beginning of the season will also be key (de Brauw et al., 2019).

The situation for GR is quite different. The impact pathways would presumably be the same as for OFSP (Huey et al., 2022), but the accelerators relating to trust, acceptance and social licence have not been fully addressed in Asia. The innovation process for GR is thus essentially stalled, largely because of opposition to transgenic crops in the region. The reasons for this are complex and partially rooted in the sociopolitical processes by which change occurs (Mueller and Flachs, 2022). The role (if any) that GR is likely to play in addressing VAD deficiency remains highly uncertain. Government approval may spur its widespread adoption in the Philippines and elsewhere in Asia, though it may not. The commercialisation of GR faces other issues too. Rice farmers may not choose to plant golden rice varieties unless they are offered specific inducements to do so (Glover et al., 2020). On the consumer side, GR needs to be integrated into a cost-effective component in broader nutrition strategies that are tailored to consumers’ socio-economic contexts (De Steur et al., 2022). Convincing farmers and consumers at scale of the value of GR may require more thoughtful and nuanced approaches to persuasion and incentivisation than hitherto seems to have been the case.

### 4.2 “Early adoption” accelerators: ECF ITM and FB

The ECF ITM and FB innovations provide contrasting examples of the “early adoption” accelerators around funding models and market incentives. The ECF ITM vaccine is an example of a sophisticated technological innovation that is still in the relatively early stages of uptake, implemented via a funding model involving a public-private partnership to provide the vaccine at market cost to small-scale livestock producers. Producing doses of vaccine is complex, time consuming and still quite expensive for livestock farmers. The vaccine also needs a well-managed cold chain, which requires infrastructure, training and coordination. Regarding incentives for vaccine use, a study in northern Tanzania showed that the size of vaccination benefits varies by household: households with larger herds see smaller improvements in food availability and diet diversity compared with households with smaller herds (Teufel et al., 2021). There are no impact data at national level yet, though ex ante estimates indicate benefit-cost ratios of about 2 associated with the use of the ECF ITM vaccine in Kenya, with modest increases in domestic supply of cattle, meat and milk and a reduction in net imports; impacts on nutrition and food security are relatively small (Toye et al., 2020). The direct impacts to date of the ECF ITM vaccine have not been as large as might have been expected (Perry and Dijkman, 2019), and its future is difficult to foresee. It remains an effective method of controlling a devastating livestock disease (Bishop et al., 2020), although its uptake may exacerbate social imbalances between men and women, and wealthier and poorer households, which would need to be guarded against (Homewood et al., 2006; Jumba et al., 2020). Other methods of addressing ECF are being worked on, and the next few years may see one being developed with fewer challenges to its roll-out at scale, particularly for the highly vulnerable livestock keepers who could benefit from it most (Nene et al., 2021).

Details of the FB innovation process are not that clear, though Mohamed-Saleem and von Kaufmann (1995) provide some information. The groundwork for the innovation was effectively carried out 40 years ago. The basic design as formulated by the researchers involved was changed in response to testing with farmers – the area fenced was often reduced, for example. The innovation was able to provide both dry-season feed for cattle and increased crop yields for adopters, but there were major constraints to its wider uptake associated with lack of know-how and relatively high costs of establishment (seed, fencing and labour). The research work appears to have included little interaction with the private sector to increase the availability and lower the cost of seeds and fencing materials (accelerators 4 and 5), and little focus on identifying and working with government and other partners for moving towards impact at scale (accelerators 6–8). The uptake of the FB innovation has not progressed much since the work was abandoned in the early 1990s. The challenge of dry-season feed resources in West Africa is as great as ever, and Amole et al. (2021) identify FBs as one of a range of strategic interventions that could be implemented to improve animal nutrition in the region, as well as contributing to land restoration. There could thus still be a significant role that FBs could play in the region, through addressing the market and policy accelerators to move the innovation to the next stages of impact in what is a highly dynamic farming environment.

### 4.3 “Moving to scale” accelerators: PICSA and SSU

Both PICSA and SSU have had considerable impact, although they provide some contrasts in the attempt to move to broader uptake and impact. The experience of PICSA in Rwanda shows that sustainable implementation at scale requires several things. These include integration into government policy and support from the intermediaries and service providers who support smallholder farmers (accelerators 5 and 6). It also requires a tight control of the quality and integrity of the approach, coupled with a robust monitoring and evaluation process (accelerator 7) to track the uptake and effectiveness of the approach (Clarkson et al., 2022). In addition, sustainable implementation models are needed that are appropriate for other countries in which PICSA is being rolled out (accelerator 8). These models are likely to require the bundling of climate services with other products such as insurance and market information that can increase the resilience and food security of smallholder farmers. Impact studies indicate strongly positive, if variable, returns on research-and-development and farm household investment in climate service provision (Thornton et al., 2023). Currently, the key gaps for an innovation such as PICSA to reach its potential impact revolve around viable and sustainable implementation plans, supported by a mix of public and private finance. The elements of such implementation plans would include the partners, scientific backstopping and communication mechanisms appropriate for different contexts and countries.

Much of the impact of SSU can be attributed to its theory of change, built around addressing both the supply and demand sides of agricultural information provision, utilising robust scientific information provided by experts, and trying to ensure that the audience identifies and empathises with the household on screen through a sharing of aspirations. Outcomes feed into the ongoing interactions among the audience, and audience requests to the production team for further information reinforces and extends learning and outcomes (Clarkson et al., 2018). Indeed, models such as SSU can be seriously considered as a viable and cost-effective means to introduce changes in farming methods that can contribute to national food security and poverty alleviation goals (Areal et al., 2020). In 2022 SSU started airing in Zambia and by the end of the series was reaching 3 million viewers through the national broadcaster ZNBC, and there are plans to expand to other countries. The rapidly evolving IT systems in many countries in the region are greatly facilitating farmers’ access to digital platforms for banking, information, marketing and inputs, provide growing opportunities for edutainment like SSU and its linkage with new products and services. These opportunities include increased collaboration with the private sector, to help foster food system change. Robust impact assessments that demonstrate sustained farm-level benefits to incomes and household well-being as a result of SSU, and close ties with national extension systems (accelerators 6–8) could greatly facilitate further applications of the SSU model in many other countries of Africa.

## 5 Discussion

The case studies present a range of situations with respect to uptake and impact. OFSP has had considerable impact, and with mainstreaming in more countries, allied with appropriate seed supply, could go to much broader scale. GR is an example of a mature technology that has no social licence to operate in most countries in Asia. Its uptake is in the hands of national governments, to legislate for its use in-country, and of rice producers and consumers, to be willing to grow and eat it. Whether GR will be able to out-compete other less contentious methods of addressing VAD is unknown. ECF ITM vaccination is another mature innovation but is fairly costly and difficult to implement. There is some uptake but far below its potential, and currently it is not reaching the people who could benefit the most from its use. There are other ways in which to control ECF, and options in the pipeline may be able to address the disease challenge more easily than ECF ITM. The FB is an innovation that seems to have languished for decades, largely because of a lack of attention given to the accelerators of change that could lead to broad impact. It could still fulfil a niche role in the livestock systems of West Africa and its time may come. PICSA has had strong impact and could go to scale if mainstreamed more broadly and with close attention to monitoring to preserve information quality. SSU is a highly promising model of how agricultural extension can operate effectively at relatively low cost, using existing communication media (TV and SMS). Development of different expansion models could take this innovation to even broader scale in different countries.

Taken together, the case studies illustrate several points. First, all have long time horizons. SSU and PICSA have the shortest, at around 10 years. The other innovations have taken 30–40 years from their original development, and their uptake has been variable. This accords with much of the historical literature (Muhammed et al., 2018; Hall and Dijkman, 2019; Krishna et al., 2023). Most examples of rapid uptake of innovation seem to be associated not with technological change per se but with regulatory and policy changes, such as occurred in the Thai poultry sector in the early 2000s as a result of avian influenza (Otte et al., 2008). This underscores the need for patience on the part of both researchers and funders. At the same time, this is deeply concerning: food system innovations that are not already well advanced now may have little impact by 2030, given the current discourse around the Paris Agreement and attainment of the Sustainable Development Goals.

Second, the case studies illustrate some of the various ways in which innovations that “work” can fail to be adopted. Golden Rice is currently stalled, mostly because of a lack of social licence to operate, though this could change in time. Fodder Banks have been stalled for decades because of lack of stable finance, lack of market incentives, and lack of public and private support. Uptake of the ECF vaccine has been hampered by cost issues and inconsistent government support.

Third, a corollary of the second point above, there appears to be only one way to succeed: major contributions are needed across all eight accelerators if uptake at scale is to occur. This is somewhat consistent with the scaling readiness approach (Sartas et al., 2020), which highlights that an innovation package is limited by its weakest or least ready component. Of the case studies, OFSP, PICSA and SSU have all had strong impact in the past, though all three currently appear to lack clear transition pathways or models for expansion in scope and reach that could result in sustainable, transformative change in the future.

Fourth, while all the accelerators appear to be needed, they are not necessarily all needed at the same time. As is reflected in Table 2, the accelerators needed depend on the stage of the innovation process that has been reached. This is borne out by recent studies on the complexity of adoption decisions, which highlight that farmers have different needs at different stages in the process (Hermans et al., 2021; de Oca Munguia et al., 2021). Just because an innovation is stuck somewhere along the impact pathway does not mean that it is necessarily stuck for ever: Golden Rice could (and may) still take off in Asia, with attention being paid to the three accelerators associated with “laying the groundwork” and safeguarding against undesirable risks. The same could potentially happen with Fodder Banks in West Africa, with respect to the “early adoption” and “moving to impact” accelerators.

Three of the accelerators score low to moderate across all or most of the case studies. It could be expected that the challenges around the availability of stable finance would be resolved for long-established and well-adopted innovations. This does not (yet) apply to any of the six innovations considered here. In the shorter term, given the time lags associated with their uptake, the need for stable finance to spur uptake is likely to be common across most innovations, involving a wide range of financing mechanisms (Cosgrove et al., 2023). For all six case studies, the policy environment is contributing only moderately (at best) to innovation uptake. Technological innovation may often precede the establishment of a policy environment that can enable uptake at scale. Existing policies may not necessarily align with the food system challenges of the future: for example, the Common Agricultural Policy of the European Union strongly supports robustness of farming systems, but there is much less focus on the adaptations that will be needed in the coming years (Buitenhuis et al., 2020). This is likely to be a common issue, except for situations where policy change itself is driving rapid system change (as in the Thai poultry sector example above). Similarly, transition pathways are notable for their weakness across all six case studies. This is reflective of a paucity of studies that address the incorporation of disruptive innovation in ways that address poverty, equity and food security issues during the transition from one food system state to another (Kerr et al., 2022); Cradock-Henry et al. (2020) is one of only a few examples.

Our application of the change accelerators of Herrero et al. (2020) to a set of historical case studies suggests to us that the framework is useful for helping to explain the fate of different innovations and why some go to scale and others do not. The framework is not without its weaknesses. One is, there is no guarantee that an innovation will go to scale, even if all the accelerators are being addressed during the innovation process. Technological innovations may become obsolete, either by being superseded by better or more cost-effective technology – this could be the fate of the ECF ITM vaccine, for example - or by a wholesale change of the socio-technical regime that renders existing technologies obsolete. An example of the latter would be an innovative, chemical herbicide developed for conventionally-managed crop production systems that have switched to organic management. Another weakness is that the accelerator framework is also not able specifically to address the issue of competing technologies. Nothing is explicitly said about the cost-effectiveness of alternative technologies that achieve the same outcome. For example, there are several ways in which VAD can be reduced, not just via OFSP and GR. These include diet supplementation, poverty alleviation, and increased dietary diversity. There are few direct comparisons of the performance of the different methods of reducing VAD in the literature, however. A further weakness is the somewhat subjective method used to estimate the contributions of the accelerators to each innovation. The robustness of these scores could be strengthened in future by defining more objective contribution indicators for each accelerator, perhaps drawing on the considerable literature on agricultural innovation and scaling (see, for example, Spielman and Birner, 2008; Sartas et al., 2020).

There are other overarching issues that are not contained explicitly within the framework but that may be important to address. The first issue concerns equity and power dynamics, particularly with respect to the enabling social license, transforming mindsets, and building trust accelerators. There are two challenges here. One is addressing the powerful and entrenched interest groups that can perpetuate path dependencies and stifle innovation in the food system (Hall and Dijkman, 2019). Another challenge is ensuring inclusive engagement of sometimes under-represented but essential perspectives in the innovation process: women, men and young people, as well as engagement with indigenous knowledge and other belief systems. Genuinely participatory approaches are crucial. A second issue relates to research data and evidence, which underpin much of any effort but are usually critical for any design of incentives, policies, regulations, and safeguarding against undesirable effects (including innovations that may increase inequity in target populations). A third issue is the challenge of building in participatory and analytical methods in the innovation process that highlight potential undesirable effects in the future, so that these can be avoided or mitigated to the extent possible.

For these reasons, we would see most value of applying the accelerator framework in the rapid screening of innovations processes in the food system aimed at enhancing the food security and resilience of small-scale producers, identifying possible issues in the roll-out of future innovations, and for decision makers in trying to balance a portfolio of interventions at different stages along their impact pathways.

## 6 Conclusions

In this paper we applied a simple framework of eight change accelerators to six historical case studies of innovation in the food system, to test its utility in helping to explain the broad issues around the uptake or otherwise of technologies that “work” but do not necessarily go to scale. We have shown that the framework can provide a useful approach to rapidly evaluating historical innovations. We have also distilled some lessons that may be useful with respect to future innovation involving technologies that are in the pipeline or still on the drawing board. Given the nature and urgency of the food system challenge, the framework can provide a useful approach to rapidly evaluating and screening future innovations to help prioritize and guide investment towards those innovations that have very high potential for making food systems more resilient, sustainable and equitable.

Hall and Dijkman (2019) highlight the existence of “… an interconnected set of changes across multiple levels of food systems involved in transition and transformation processes” and the limitations of technology by itself to drive transformation without the accelerators in place, to create new socio-technical regimes allowing the emergence of sustainable, inclusive agri-food systems. Looking for quick technological wins seems doomed to failure; much more likely, food system change will come about via myriad complex, long-term innovation processes that can be accelerated in appropriate ways, that shift food systems in new directions that need to be agreed upon by all the food system actors operating in specific regional, national and local contexts. As persuasively argued by Turnhout and Lahsen (2022), if progress is to be made in the face of existential challenges, new science-society contracts need to be built that explicitly recognise the politics of knowledge; and research-for-development innovation needs to do its part in this.

## Declaration of competing interest

The authors declare that they have no known competing financial interests or personal relationships that could have appeared to influence the work reported in this paper.

**Annex Table A1:** Accelerators of change in the biofortified orange-fleshed sweet potato (OFSP) innovation process.

Accelerator	What was done?	Who did it?	Contribution (0–5)	What is lacking?
1 Building trust amongst actors in the food system “Vision & Values”	Demonstrating the potential of OFSP to/with the nutrition community	Ag research community, donors	5	-
2 Transforming mindsets “Acceptance”	Studies of the social acceptability of OFSP by consumers	Ag and nutrition research communities	5	-
3 Enabling social licence and stakeholder dialogue “Responsibility”	Engagement with all value chain actors and consumers	Ag and nutrition research communities	5	-
4 Ensuring stable finance “Explore & pilot”	Development of urban (and rural) demand for the long run	Ag and nutrition research communities, public sector	3	This is still in progress
5 Designing market incentives “Spread cost & risk”	Development of urban demand (education, training) and the (public) seed sector	Ag and nutrition research communities, public sector	4	Can the private sector become more involved?
6 Changing policies and regulations “Expectations of support”	Very uneven across the countries of SSA	National ag, food and health programs	2	More mainstreaming in national policies needed
7 Safeguarding against undesirable effects “Monitor & correct”	Multi-season, multi-stakeholder monitoring	R&D community	5	Is there enough evidence on the equity implications of OFSP adoption?
8 Developing transition pathways “How and when”	Not clear		2	Need to develop scaling models for local conditions and their implementation pathways

**Annex Table A2:** Accelerators of change in the Golden Rice (GR) innovation process.

Element	What was done?	Who did it?	Contribution (0–5)	What's lacking?
1 Building trust amongst actors in the food system “Vision & Values”	Demonstrating the potential of GR to producer and consumer communities	Ag research community, government	2	Much more trust needs to be built
2 Transforming mindsets “Acceptance”	Studies of the social acceptability of GR	Ag and nutrition research communities	2	Much more still to be done
3 Enabling social licence and stakeholder dialogue “Responsibility”	Engagement with all value chain actors and consumers	Ag and nutrition research communities	2	Much more still to be done
4 Ensuring stable finance “Explore & pilot”	Development of urban (and rural) demand for the long run	Public and private sectors	3	This is still in progress
5 Designing market incentives “Spread cost & risk”	Development of urban demand (education, training) and the (public) seed sector	Ag and nutrition research communities, public sector	3	Still in progress
6 Changing policies and regulations “Expectations of support”	20 years of work, one country has approved to date	National ag, food and health programs	2	Still in progress
7 Safeguarding against undesirable effects “Monitor & correct”	Nothing done yet	R&D community	0	Monitoring will be required if GR starts to take off
8 Developing transition pathways “How and when”	Not clear		1	Need to develop scaling models for local conditions and their implementation pathways

**Annex Table A3:** Accelerators of change in the ECF infection and treatment (ECF ITM) innovation process.

Element	What was done?	Who did it?	Contribution (0–5)	What's lacking?
1 Building trust amongst actors in the food system “Vision & Values”	Demonstrating the potential and viability of an ECF vaccine	National and international research communities	4	Clear articulation as to whether ECF vaccination is a private or public good
2 Transforming mindsets “Acceptance”	Demonstrating the effectiveness of an ECF vaccine	Research communities, governments	3	Possible conflicts of interest of private agro-pharma input suppliers
3 Enabling social licence and stakeholder dialogue “Responsibility”	Engagement with value chain actors on the safety of a live vaccine	Research communities	4	-
4 Ensuring stable finance “Explore & pilot”	Development of a working public-private partnership platform	Research communities, GALVmed	4	More work needed on end-to-end solutions for poor rural livestock keepers
5 Designing market incentives “Spread cost & risk”	Development of a mechanism to deliver ECF vaccine cost effectively to those who need it most	GALVmed, governments	2	This challenge has yet to be overcome
6 Changing policies and regulations “Expectations of support”	Register and licence the ECF vaccine for use in multiple countries of eastern & southern Africa	Research communities, GALVmed, governments	2	ECF vaccine registered and licensed in extremely few countries
7 Safeguarding against undesirable effects “Monitor & correct”	Monitor the adoption and impacts of ECF vaccine	R&D community	1	Little has been done and there are few details available of adoption and impacts on the ground
8 Developing transition pathways “How and when”	Develop pathways for roll-out and uptake at scale	Research communities, GALVmed, national vet services	1	Little has been done

**Annex Table A4:** Accelerators of change for the fodder bank (FB) innovation.

Element	What was done?	Who did it?	Contribution (0–5)	What's lacking?
1 Building trust amongst actors in the food system “Vision & Values”	Working with livestock farmers to understand their major constraints	Research and extension organisations	5	-
2 Transforming mindsets “Acceptance”	Demonstrations of the technology, and iterations to improve it based on farmers' testing	Research and extension orgs	3	-
3 Enabling social licence and stakeholder dialogue “Responsibility”	Assessing whether the technology was viable in different contexts, and adjusting where possible	Research and extension orgs, and farmers	3	-
4 Ensuring stable finance “Explore & pilot”	The importance was recognised, but direct action was seen as being beyond the purview of R4D		1	More work needed on engaging the private sector and governments in developing a working financial model for the technology
5 Designing market incentives “Spread cost & risk”	The importance was recognised, but direct action was seen as being beyond the purview of R4D		1	A challenge that has yet to be overcome, though public-private partnerships could potentially provide solutions
6 Changing policies and regulations “Expectations of support”	The importance was recognised, but direct action was seen as being beyond the purview of R4D		1	Working with policy makers to highlight the importance of supporting feed resources and feed marketing in the region
7 Safeguarding against undesirable effects “Monitor & correct”	Limited monitoring and evaluation activities	Research organisations	2	Little has been done in recent times and there are few details available of adoption and impacts on the ground
8 Developing transition pathways “How and when”	R4D identified the key disenablers of uptake but went no further	Research organisations	2	The technology is relatively undisruptive, and transition pathways could be designed and targeted at e.g. women livestock farmers

**Annex Table A5:** Accelerators of change in the Participatory Integrated Climate Services for Agriculture (PICSA) innovation process.

Element	What was done?	Who did it?	Contribution (0–5)	What's lacking?
1 Building trust amongst actors in the food system “Vision & Values”	Use of local trainers and radio clubs	Project personnel	5	-
2 Transforming mindsets “Acceptance”	Peer-to-peer communication	Farmers	5	-
3 Enabling social licence and stakeholder dialogue “Responsibility”	New information dissemination channels set up	Project personnel	5	-
4 Ensuring stable finance “Explore & pilot”	Not clear what is happening w.r.t financing		2	New business models need to be developed
5 Designing market incentives “Spread cost & risk”			1	Need to involve the private sector in the value chain Need mechanisms to maintain local quality of the information
6 Changing policies and regulations “Expectations of support”	Not clear what is happening		2	Need to mainstream support as part of the national extension strategy – this has been done in Malawi, but not yet in Rwanda
7 Safeguarding against undesirable effects “Monitor & correct”	Multi-season monitoring	Project personnel	3	Robust impact assessments
8 Developing transition pathways “How and when”			1	Need to develop bundles of tech (including climate services) for local conditions and their implementation pathways

**Annex Table A6:** Accelerators of change for the Shamba Shape-Up (SSU) innovation.

Element	What was done?	Who did it?	Contribution (0–5)	What's lacking?
1 Building trust amongst actors in the food system “Vision & Values”	Relying on solid science for content	Mediae^1^	5	–
2 Transforming mindsets “Acceptance”	Following a long tradition of reality TV shows/edutainment	Others	5	–
3 Enabling social licence and stakeholder dialogue “Responsibility”	Engagement with both the supply and demand side of information	Mediae, funders, research organisations	5	–
4 Ensuring stable finance “Explore & pilot”	Development of strong linkages with development funders	Mediae, funders	3	Greater diversity of funders needed for stability and sustainability
5 Designing market incentives “Spread cost & risk”	Difficult to identify the degree of engagement with the private sector	Mediae, input suppliers	3	More direct engagement on input suppliers and other value chain actors would strengthen the approach
6 Changing policies and regulations “Expectations of support”	Unclear: the innovation system largely exists		2	More direct engagement with existing extension systems could help to embed the approach more firmly
7 Safeguarding against undesirable effects “Monitor & correct”	Annual monitoring and evaluation of each series	Mediae and M&E partners	4	More robust impact assessments are needed of the lasting effects on income and food security
8 Developing transition pathways “How and when”	Developing an expansion model for SSU in other countries	Mediae	3	Unclear what the expansion model is for different countries

1. SSU is produced by the Mediae Company (mediae.org/).

## Data Availability

No data was used for the research described in the article.
